# A novel role for 12/15-lipoxygenase in regulating autophagy

**DOI:** 10.1016/j.redox.2014.11.005

**Published:** 2014-12-03

**Authors:** Alwena H. Morgan, Victoria J. Hammond, Machiko Sakoh-Nakatogawa, Yoshinori Ohsumi, Christopher P. Thomas, Fabien Blanchet, Vincent Piguet, Kirill Kiselyov, Valerie B. O’Donnell

**Affiliations:** a Institute of Infection and Immunity, School of Medicine, Cardiff University, Cardiff CF14 4XN, UK; b Frontier Research Center, Tokyo Institute of Technology, Nagatsuta 4259-S2-12, Yokohama, Japan; c Department of Biological Sciences, University of Pittsburgh, Langley Hall, 4249 Fifth Avenue, Pittsburgh, PA 15260, USA

**Keywords:** Lipid, Autophagy, Lipoxygenase, Mitochondria

## Abstract

12/15-Lipoxygenase (LOX) enzymatically generates oxidized phospholipids in monocytes and macrophages. Herein, we show that cells deficient in 12/15-LOX contain defective mitochondria and numerous cytoplasmic vacuoles containing electron dense material, indicating defects in autophagy or membrane processing, However, both LC3 expression and lipidation were normal both basally and on chloroquine treatment. A LOX-derived oxidized phospholipid, 12-hydroxyeicosatetraenoic acid-phosphatidylethanolamine (12-HETE-PE) was found to be a preferred substrate for yeast Atg8 lipidation, versus native PE, while both native and oxidized PE were effective substrates for LC3 lipidation. Last, phospholipidomics demonstrated altered levels of several phospholipid classes. Thus, we show that oxidized phospholipids generated by 12/15-LOX can act as substrates for key proteins required for effective autophagy and that cells deficient in this enzyme show evidence of autophagic dysfunction. The data functionally link phospholipid oxidation with autophagy for the first time.

## Highlights

• 12/15-Lipoxygenase-deficient macrophages show evidence of autophagic dysfunction.
• 12-HETE-PE is a substrate for LC2 and Atg8 lipidation.
• Macrophages deficient in 12/15-lipoxygenase show altered phospholipid content.

## Graphical abstract

**Figure:**
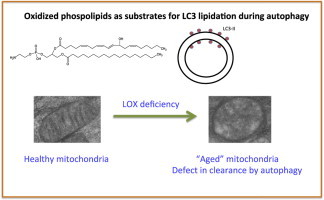

## Introduction

Murine 12/15-lipoxygenase (LOX) and its human homolog 15-LOX have long been known as generators of free acid eicosanoids, primarily 12- and 15-hydroxyeicosatetraenoic acids (HETEs), respectively. More recently, we showed these enzymes directly oxidize intact phospholipid, generating phosphatidylethanolamine (PE)-esterified forms that can dampen Toll-like receptor 4 signaling in human monocytes [1,2]. Analogous lipids are generated by neutrophil 5-LOX and platelet 12-LOX, including phosphatidylcholine (PC) esterified homologs that can stimulate coagulation and regulate leukocyte anti-bacterial actions [3,4]. Since HETE-PEs remain cell associated following their generation, we sought to examine whether they could be involved in membrane regulatory processes.

Autophagy is the process by which cells remove ageing organelles and damaged cellular structures [5]. There are three defined types of autophagy: macro-, micro-, and chaperone-mediated, all of which promote proteolytic degradation of cytosolic components at the lysosome. Autophagy begins with an isolation membrane, also known as a phagophore that is likely derived from lipid bilayer contributed by the endoplasmic reticulum (ER) and/or the trans-Golgi and endosomes. This expands to engulf intracellular cargo, sequestering it in a double-membraned autophagosome. This matures through lysosome fusion, promoting degradation of autophagosomal contents by lysosomal hydrolases. Lysosomal permeases and transporters export amino acids and other by-products of degradation back out to the cytoplasm, where they are re-used for cellular processes [6]. One particular type of autophagy, mitophagy, which removes old and damaged mitochondria, comprises several different processes termed Types 1–3 [7]. Several recent studies have highlighted a role for redox changes being pre-requisite or closely associated with autophagy, including elevated oxidative stress, lipofuscin formation, activation by terminal lipid oxidation products and changes in cellular thiol status [8–11]. During phagophore formation, lipidation of cytoplasmic LC3-I to LC3-II by conjugation with PE is considered an essential event [12]. Currently, the specific PEs that conjugate with LC3 in mammalian cells are not known, although di-oleoyl-PE is commonly used as substrate with recombinant LC3 and its yeast homolog, Atg8 [13]. Of relevance to this, 15-LOX is induced during reticulocyte maturation where it was proposed to play a role in degradation of intracellular organelles, specifically mitochondria [14,15]. During this, high levels of the enzyme are induced and cellular membranes contain detectable levels of oxidized lipid. Mitochondrial degradation has been shown to be reliant on the expression of 15-LOX in reticulocytes, with a spike in 15-LOX expression immediately before organelle degradation. It’s been shown that 15-LOX integrates into the membranes of organelles, allowing release of proteins from the organelle lumen and access of proteases to both lumenal and integral membrane proteins [15]. Whether LOXs are involved in autophagy or other membrane processing events is currently unknown, although previous studies have shown that 12/15-LOX-deficient cells show defective phagocytosis linked to altered actin polymerization in mice [16].

In this study, we examine membrane ultrastructure and LC3 expression and lipidation in macrophages from mice lacking 12/15-LOX, and determine the ability of oxidized phospholipids to act as substrates for LC3 lipidation in vitro. The results suggest a role for the pathway in regulating dynamic membrane alternations in mammalian cells.

## Materials and methods

### Isolation of mouse macrophages

All animal experiments were performed in accordance to the United Kingdom Home Office Animals (Scientific Procedures) Act of 1986. C57BL/6 wild type (from Charles River) and 12/15-LOX^−/−^ mice (8–12 weeks) were kept in constant temperature cages (20–22 °C) and given free access to water and standard chow, and killed using CO_2_ asphyxiation. Peritoneal lavages were carried out using 2 ml PBS. Lavages were pooled, pelleted by centrifugation and re-suspended in media (RPMI media, 10% (v/v) fetal bovine serum, 100 µg/ml penicillin, 100 µg/ml streptomycin, and 2 mM glutamine). Cells were either used directly or seeded in flasks at 100×10^6^ cells/ml to isolate the macrophages, by adhesion (2 h at 37 °C). Macrophages washed once with RPMI media, fresh monocyte media was added to the flasks and the macrophages were then released by gentle scraping. Macrophages were pelleted as described above, washed and pelleted in PBS, re-suspended in Krebs buffer, counted, and diluted to 4×10^6^ cells/ml for experiments.

### Transmission electron microscopy of macrophages

Murine macrophage pellets were submerged in cacodylate buffer containing 2.5% glutaraldehyde and stored at 4 °C up to 4 weeks. Samples were washed twice for 15 min with 0.1 M cacodylate buffer then re-suspended in 1% osmium tetroxide in 0.2 M cacodylate buffer and incubated at 21 °C for 1 h. Samples were washed 4× for 15 min with H_2_O then stained with 0.5% uranyl acetate in dH_2_O for 1 h at 21 °C. Samples were dehydrated by re-suspending in increasing percentages of ethanol, for 15 min each: 50%, 70%, 80%, and 90% followed by 3 times with 100% ethanol. Samples were transferred to glass vials and re-suspended in propyl oxide. Resin infiltration was carried out by re-suspending samples in 1:1 pre-mixed embedding resin and propyl oxide overnight, at room temperature, leaving vials open. Cell samples were immersed further with fresh embedding resin and transferred into plastic molds. Cell pellets were allowed to settle, following 2 h at 21 °C, samples were transferred to 60 °C for 48 h. 90 nm sections were cut from 3 different pellet locations using a Reichert-Jung Ultracut E microtome. Sections were mounted onto naked grids which were stained using 2% uranyl acetate for 10 min, washed twice with distilled water followed by staining with Reynold's lead citrate for 5 min and an additional two washes with dH_2_O. Samples were dried on filter paper then analyzed by transmission electron microscopy, on a Philips EM208. Kodak EM 2289 film (Agar Scientific, Stansted, Essex, UK) were developed for 3.5 min, at 20 °C in Kodak D-19 developer, diluted 1:2 with H_2_O. Films were fixed for 30 s in an acetic acid, followed by 4 min in Ilford Hypam fixer, diluted 1:3 with H_2_O, rinsed then dried.

### Phospholipid profiling of macrophage lipids

Macrophages were suspended in 0.5 ml Krebs buffer and the lipids extracted using 1 M acetic acid:2-propanol:hexane (2:20:30) containing internal standards (10 ng/ml sample volume, listed below), and extracted as previously described [1]. Extracts were suspended in methanol and stored at −70 °C until analysis. Phospholipids were profiled by LC/ESI/MS/MS on a 4000 Q-Trap (AB Sciex, Warrington). Phospholipids were separated using 50–100% B over 10 min then 100% B for 30 min at 200 µl/min (A is the methanol:acetonitrile:water at 6:2:2 with 1 mM ammonium acetate; B is the methanol with 1 mM ammonium acetate), using the specific parent to daughter transitions shown in Supplementary Tables 1–6. Relative levels of lipids were determined by comparison to internal standards with the following parent to daughter transitions *m/z* 634–227 (DMPE) [M−H]^−^, 678–184 (DMPC) [M+H]^+^, 591–227 (DMPA) [M−H]^−^ and 665–227 (DMPG) [M-H]^−^. PS-phospholipid profiling was carried out by flow injection using the phospholipid solvent system running at 50:50 A:B, 1 ml/min for 6 min. Products were profiled using an internal standard, with parent to daughter transition of *m/z* 678–227 (DMPS) [M−H]^−^.

### Cholesteryl ester profiling of macrophage lipids

Precursor mass spectra were obtained operating in positive mode. Samples were introduced at 10 µl/min in methanol using a Hamilton syringe. The de-clustering potential and collision energy were −140 and −45 V respectively. Spectra were obtained from *m/z* 100 to 1000 amu over 12 s with 10 MCA scans acquired. Cholesteryl esters were then detected by LC/MS/MS, having adapted a method described by Ferreira et al. [17]. Cholesteryl esters were separated on a C_18_ ODS2, 5 µM, 150×4.6 mm^2^ column (Waters Ltd., Elstree, Hertfordshire, UK) using an isocratic method with mobile phase propan-2-ol:acetonitrile:ammonium acetate (60:40:4) at 1 ml/min. Products were profiled by LC/ESI/MS/MS using the specific parent to daughter transitions of *m/z* 668, 666, 682, 690, 706, 642, 640, 670,708, 714 and 730–369.1 (cholesterol) ([M+NH_4_]^+^) (Supplementary Scheme 1). The collision energy for cholesteryl esters was −33 V and the declustering potential, −91 V.

### Inhibition of autophagy post-initiation and Western blotting of LC3-I and -II

Murine peritoneal macrophages were isolated from male WT and 12/15-LOX^−/−^ mice and cells from two mice from each group were pooled. 9×10^5^ cells were incubated in a 24 well plate with and without chloroquine (100 µM) for 20 h. Supernatants were removed and cells washed gently with PBS twice to remove serum. Cells were lysed in 50 µl lysis buffer (Stock: 200 µl 2% Ipegal CA-630, 40 µl 0.5 M EDTA, 1 ml 1.5 M NaCl, 100 µl 1 M Tris–CL, 0.5% (w/v) sodium deoxycholate, and 8.46 ml distilled water), 100 µl 10× protease inhibitor cocktail on ice for 15 min, followed by vortexing and further 10 min incubation on ice. Lysates were then centrifuged for 15 min at 13,000 rpm and supernatants removed to new tubes. Lysates were reduced and boiled at 80 °C for 10 min. Protein concentration was quantified using a BCA test to ensure equal loading. Protein extracts were separated by SDS-PAGE using a gradient polyacrylamide gel (4–12%) (Invitrogen), and subsequently transferred to a 0.45 µM nitrocellulose (Amersham™ Hybond ECL, GE Healthcare, Life Sciences). Membrane was blocked for 1 h in PBS/0.05% Tween/5% milk, and then probed overnight with a polyclonal anti-mouse LC3 (1 µg/ml) (sigma L8918) and subsequently an anti-mouse actin (clone C4, Millipore, Temecula, CA92590, and MAB1501R), in PBS/0.05% Tween/1% BSA. Blot was then probed with a polyclonal goat anti-rabbit coupled to HRP (Dako (PO448)) and incubated with ECL (Pierce). Blot was exposed for 1 min onto x-ray film.

### Enzymatic lipidation of Atg8 and LC3

All proteins were purified from *Escherichia coli*. However, purified LC3B, hs (*Homo sapiens*) Atg7, and hsAtg3 are kind gifts from Nobuo N. Noda, Institute of Microbial Chemistry, Tokyo 141-0021, Japan. In vitro lipidation reactions of Atg8 and LC3 were performed using buffer containing 50 mM Tris–HCl pH 8.0, 100 mM NaCl, 1 mM MgCl_2_, and 0.2 mM DTT. Purified Atg7 (1 µM), Atg3 (1 µM), and Atg8 (5 µM) were incubated at 30 °C with liposomes (350 µM) composed of 55 mol% PE (di-oleoyl: DOPE, 1-stearoyl-2-arachidonyl: SAPE, or HETE-PE), 35 mol% 1-palmitoyl-2-oleoyl-phosphatidylcholine (POPC), and 10 mol% yeast phosphatidylinositol (PI) in the presence of 1 mM ATP for the indicated time periods, followed by urea-SDS-PAGE and CBB-staining. Purified hsAtg7 (1 µM), hsAtg3 (2 µM), and LC3 (5 µM) were incubated at 37 °C with liposomes (350 µM) composed of 55 mol% PE, 35 mol% POPC, 10 mol% yeast PI or 10 mol% PE, 80 mol% POPC, 10 mol% yeast PI in the presence of 1 mM ATP for the indicated time periods, followed by SDS-PAGE and CBB-staining.

## Results

### Macrophages deficient in 12/15-LOX show altered ultrastructure

**Fig. 1:**
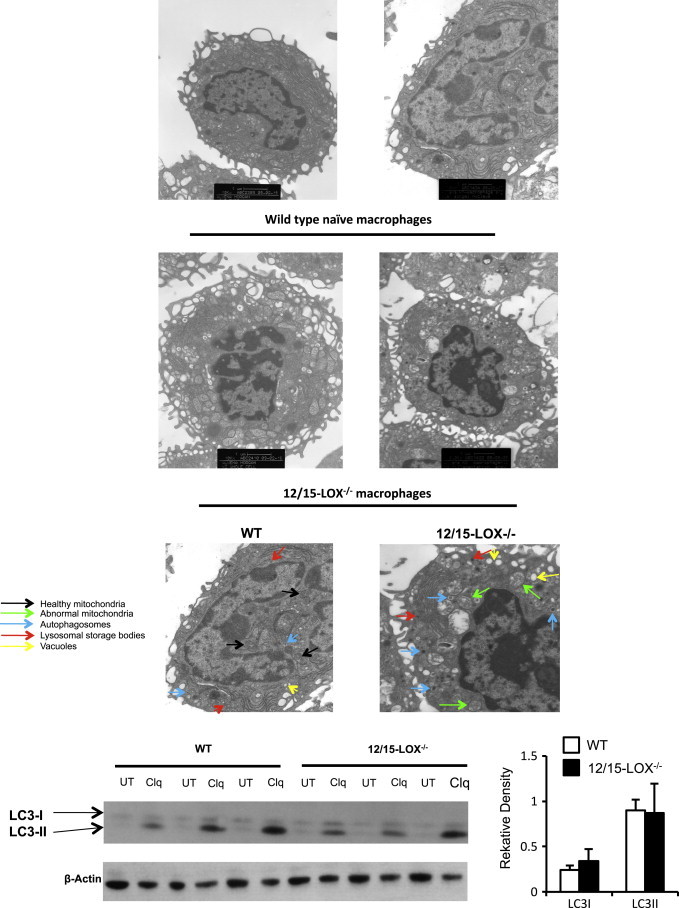
Macrophages from 12/15-LOX deficient mice show altered membrane structure on electron microscopy but LC3 expression is similar. Panel A. EM analysis of wild type and 12/15 LOX^−/−^ peritoneal macrophages. Peritoneal cells from wild type mice were analyzed using TEM as described in Section “Materials and methods” at 10,000× magnification. Lower panels. Cells were analyzed by TEM at 20,000× magnification. Arrows indicate healthy mitochondria (in WT) (black), abnormal mitochondria (in 12/15-LOX^−/−^) (green), and numerous autophagosomes (blue), lysosomal storage bodies (red) and vacuoles (yellow). Panel B. Macrophages from 12/15-LOX^−/−^ mice express similar LC3 levels to wild type. Macrophages were stimulated overnight using chloroquine (100 µM), before LC3-I and -II analysis using Western blot. Data are combined from three representative gels, with one shown as illustration. Each gel had *n*=3 for both WT and 12/15-LOX^−/−^ mice. Relative density was determined for LC3-I and -II, then divided by the actin loading control density, thus the graph represents a combined *n*=9, mean±SEM.

Peritoneal cells from naïve mice were analyzed using transmission EM. Representative macrophages from three separate pooled isolates is shown in Fig. 1A. Healthy-looking mitochondria (small, compact, and with well-defined cristae) are seen in wild type cells. In contrast, 12/15-LOX^−/−^ macrophages are swollen and granular. 12/15-LOX^−/−^ macrophages also demonstrate a large number of vacuoles (yellow arrows) and potential lysosomal storage bodies, visible as dark inclusions (red arrows). Some have double membranes, suggestive of autophagosomes (blue arrows). Far lower numbers of vacuoles and suspected lysosomal storage bodies are seen in wild type macrophages.

### LC3 expression and lipidation in 12/15-LOX-deficient macrophages is unchanged

Macrophages from both WT and 12/15-LOX^−/−^ mice show low levels of LC3-I and II by the Western blot. To inhibit the turnover of autophagosomes, cells were incubated with chloroquine, which raises the lysosomal pH, and leads to inhibition of both fusion of autophagosome with lysosome and lysosomal protein degradation. As a result, we see an accumulation of LC3-II which is the membrane associated lipidated form. Macrophages from 12/15-LOX^−/−^ mice contained similar amounts of LC3-I and LC3-II to wild type controls, although there was a high degree of variability between mice (Fig. 1B).

### 12-HETE-PE is an effective substrate for lipidation of both Atg8 and LC3

**Fig. 2:**
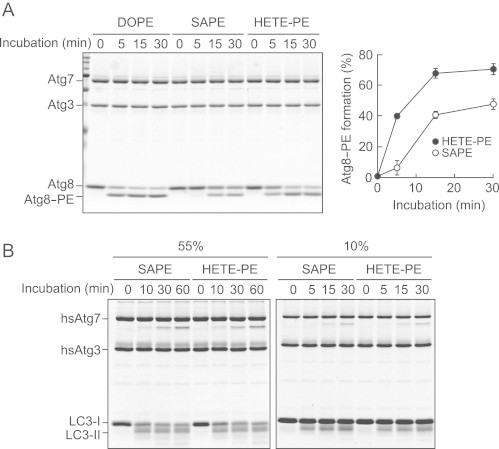
Atg8 and LC3 can be conjugated to 12-HETE-PE. Panel A. Atg7, Atg3, and Atg8 were incubated with liposomes composed of 55 mol% PE (DOPE, SAPE, or HETE-PE), 35 mol % POPC, and 10 mol% yeast PI, with 1 mM ATP for the indicated time periods, followed by urea-SDS-PAGE and Coomassie brilliant blue (CBB)-staining. Efficiency of Atg8 lipidation was calculated by dividing the intensities of Atg8-PE by those of total Atg8. The graph shows results of three independent experiments (mean±SD, ^⁎⁎^*p*<0.01, ^⁎⁎⁎^*p*<0.001, HETE-PE versus SAPE, Student's *t*-test). Panel B. Purified hsAtg7, hsAtg3, and LC3 were incubated at 37 °C with liposomes composed of 55 mol% PE, 35 mol % POPC, 10 mol% yeast PI (left panel) or 10 mol% PE, 80 mol% POPC, 10 mol% yeast PI (right panel) in the presence of 1 mM ATP for the indicated time periods, followed by SDS-PAGE and CBB-staining.

To examine whether Atg8 is conjugated to HETE-PE or SAPE, in vitro conjugation reactions using liposomes composed of mixed PE/PC and yeast PI, where the PE consisted of DOPE, SAPE or 15-HETE-PE, were undertaken. DOPE is shown for comparison, as this is the usual lipid used for Atg8 conjugation reactions, rather than SAPE [18]. As shown in Fig. 2A, Atg8 was conjugated to HETE-PE more efficiently than SAPE. In addition, the mobility of Atg8-HETE-PE/SAPE and Atg8-DOPE was different, specifically the mobility of Atg8-HETE-PE/SAPE was slightly lower than that of Atg8-DOPE. This is likely due to the longer fatty acid chain length at the *sn2* position of SAPE/HETE-PE. A comparison of SAPE-PE versus HETE-PE was conducted three times, and densitometry scanning averaged, clearly showing HETE-PE as a preferred substrate at all time points tested versus SAPE (Fig. 2A, right panel). This indicates that oxidized phospholipids can be conjugated to Atg8, and that introduction of the –OH at C15 leads to a more effective substrate.

**Fig. 3:**
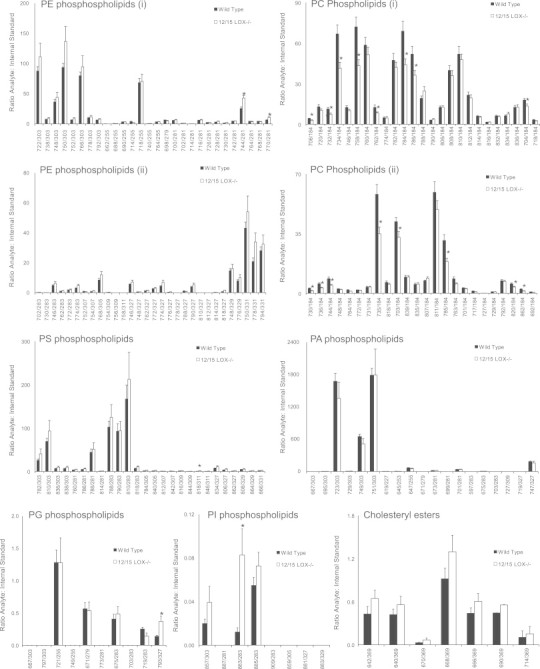
Lipidomic profiling reveals altered phospholipid and cholesteryl esters in 12/15-LOX deficiency. Lipids were extracted from macrophages, and analyzed as described in Section “Materials and methods” (*n*=8, mean±S.E.). *Student's *t*-test, *p*<0.05. The overall differences between WT and 12/15-LOX data sets is significant following analysis by one-way ANOVA with a Tukey' post-hoc test, *p*<0.05 (except for cholesteryl esters).

Next, the ability of HETE-PE to act as a substrate for the mammalian LC3 was tested using recombinant proteins. In these experiments, it was initially seen that 55 mol% SAPE and HETE-PE were similarly conjugated over 30 min (Fig. 2B, left panel). Thus, we also tested a lower substrate concentration (10 mol% PE) and shorter time course, in case the enzyme system was already saturated, but no differences were found between the lipids (Fig. 2B, right panel). DOPE conjugation to LC3 is shown as comparison (Fig. 3B). The results demonstrate that oxidized PE is an effective substrate for LC3 lipidation, although in this case, it is equally effective as the unoxidized parent lipid.

### Macrophages deficient in 12/15-LOX show modified cellular phospholipid content

To examine for changes in cellular lipid profiles resulting from 12/15-LOX deficiency, lipidomics profiling of all phospholipid classes and cholesteryl esters was undertaken on lipid extracts from macrophages obtained from naïve wild type and 12/15-LOX^−/−^ macrophages. There was a tendency overall for increased PE, PI and cholesteryl esters, but decreased PC in 12/15-LOX deficiency. On the other hand, PA, PS and PG were not different. This suggests that loss of the enzyme results in a selective defect in particular phospholipid classes at the expense of others (Fig. 3).

## Discussion

Herein, we show that deficiency of the lipid-oxidizing enzyme, 12/15-LOX, is associated with altered cellular membrane structure. We also demonstrate that a LOX-derived oxidized phospholipid is an effective substrate for lipidation of both LC3 and Atg8, being preferred over the unoxidized analog in the case of the yeast homolog. This is suggestive of this pathway being involved in regulation of membrane dynamics. Last, we show altered phospholipid content in murine macrophages deficient in 12/15-LOX. Our observations of double membrane structures suggestive of autophagosomes propose a role in autophagy. Normal LC3 expression and lipidation indicate that the defect in the 12/15-LOX^−/−^ macrophages is likely to be upstream of LC3 activity itself.

12/15-LOX was first described as the human homolog, 15-LOX1, as being highly induced in bleeding anemia in rabbits, inducing significant peroxidation of intracellular membranes that coincided with disappearance of organelles [19–23]. Thus, it was proposed as being critically required for reticulocyte maturation into erythrocytes. However subsequent to this, mice deficient in the functional homolog, 12/15-LOX were shown to have normal red cell counts, and interest in this pathway waned [24]. This does not exclude that the knockout mice have developed a compensatory mechanism, and that the enzyme still plays a role in normal turnover of organelles during homeostasis. In support of a role for LOX in processes that involve membrane remodeling, previous studies have shown that 12/15-LOX^−/−^ macrophages are unable to undergo a full phagocytosis response towards apoptotic thymocytes [25].

The multiple differences between wild type and 12/15-LOX^−/−^ macrophages seen, including abnormal mitochondria, multiple lysosomal storage bodies and suspected autophagosomes are consistent with LSDs [26–29]. Lysosomes are small vesicular organelles, their primary function being to merge with late endosomes to digest their content [30–32]. Endosomal degradation is carried out by numerous lipid and protein hydrolases. Mutations in these can cause build-up of undigested cellular content seen as dark inclusions, and similar structures were seen herein in 12/15-LOX^−/−^ macrophages [30,33].

Lysosomes participate in autophagy, required for rapid clearance of oxidized proteins and organelles [34,35]. Both lysosomes and autophagy are important regulators of mitochondrial turnover, with those in 12/15-LOX^−/−^ macrophages appearing swollen and granular, suggesting they are ‘old’ and damaged, and should have undergone autophagy. The phenotype of cells showing signs of LSD resembles that of aged cells, with abnormal mitochondria and lysosomal storage bodies [30]. There are several common dysfunctions leading to LSDs, including of relevance, the mutation in glucocerebrosidase (Gaucher's disease) where the lipid glucosylceramide accumulates in several cells, and is characterized by macrophages containing high levels of lysosomal lipid [36]. Of relevance, splenomegaly is also a feature of Gaucher’s disease, also previously observed in mice with 12/15-LOX^−/−^ deficiency [37].

Preventing autophagy leads to mitochondrial damage to the cells due to oxidative stress [38]. A progressive increase in autophagic vacuoles is in accordance with disproportionate organelle damage and degradation, recognized as ‘autophagic stress’, and is consistent with the phenotype of 12/15-LOX^−/−^ macrophages seen herein [39]. In this study, autophagosomes were seen as inclusions with double membranes (Fig. 1). Primary LSDs are commonly associated with ‘swirls’ in cells, but they were not present in 12/15-LOX^−/−^ macrophages [40]. This suggests that the dark inclusions, identified as storage bodies, are not the primary storage compartment for this undigested material.

LC3 and its yeast homolog Atg8 are considered important markers and effectors of autophagy, undergoing covalent linkage of the C-terminus to the PE headgroup, leading to anchoring on the cytoplasmic and luminal sides of autophagic vesicles. Currently, the identity of the specific molecular species of PE that are conjugated to LC3/Atg8 are unknown and herein our observation that HETE-PE can be conjugated to these proteins, and indeed is a preferred substrate in the yeast system, functionally links phospholipid oxidation with autophagy for the first time (Figs. 2 and 3). We note that levels of LC3-I and -II appeared normal in 12/15-LOX^−/−^ mice however, suggesting that the defect in these cells is upstream of this protein. 12/15-LOX generates oxidized phospholipids that remain cell associated in macrophages, including derivatives that contain reactive carbonyl groups termed keto-eicosatetraenoic acid-PEs (KETE-PEs) [41]. We previously showed these can form Michael adducts with proteins, and herein, that one of them is an effective substrate for LC3 lipidation [41] (Fig. 1). Thus, the absence of these in the knockout could lead to loss of function of key autophagy proteins, required for effective clearance of aged organelles. We did not examine in this study whether other LC3 family members, for example GATE-16, GABARAP, or GABARAPL1 could also act as substrates for HETE-PE conjugation. Exactly how 12/15-LOX deficiency results in altered lysosomes is also not known and will be the subject of future studies.

Interestingly, mice deficient in 12/15-LOX are generally healthy, only showing a phenotype when challenged (protected against several inflammatory diseases) [42,43]. As 12/15-LOX and its human homolog 15-LOX is only expressed in selected immune cells, including resident macrophages, Th2-cytokine challenged monocytes, eosinophils and also epithelia, a role in specialized autophagy-related processes is more likely. In the case of macrophages, this would include phagocytosis, recently shown to also involve the autophagy machinery, including LC3 [44].

In summary, this study demonstrates that deficiency in 12/15-LOX results in a lysosomal storage disorder phenotype, impacting on membrane processing, organelle clearance and autophagy in murine macrophages. The ability of oxidized phospholipids to act as LC3/Atg8 lipidation substrates links phospholipid oxidation, a key event in innate immunity and atherosclerosis with normal cellular processes required for cellular turnover and homeostasis.
