# Supplementary figures and images for: A novel role for 12/15-lipoxygenase in regulating autophagy
Source: Redox Biol. 2014 Dec 3;4:40–7. doi: 10.1016/j.redox.2014.11.005 (PMC4309860; doi:10.1016/j.redox.2014.11.005)

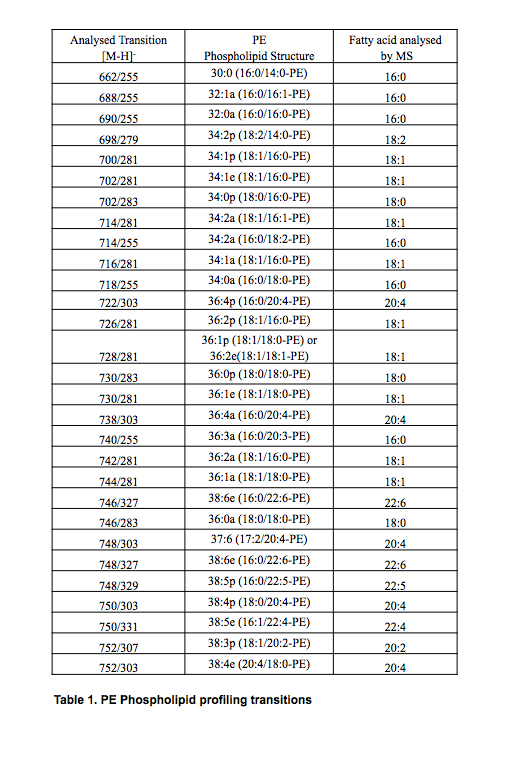


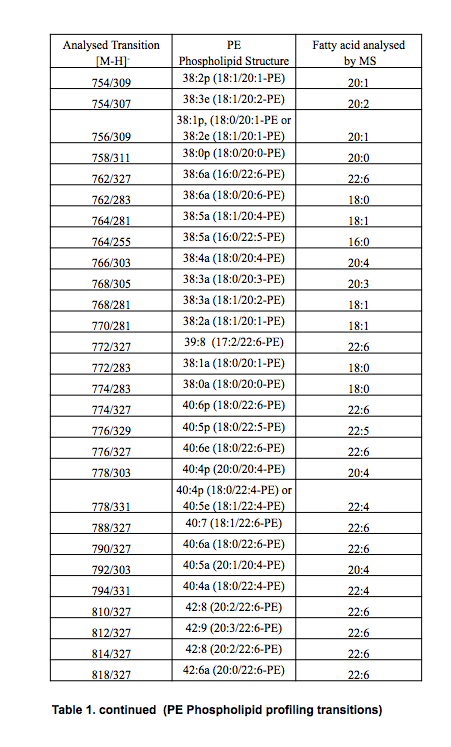


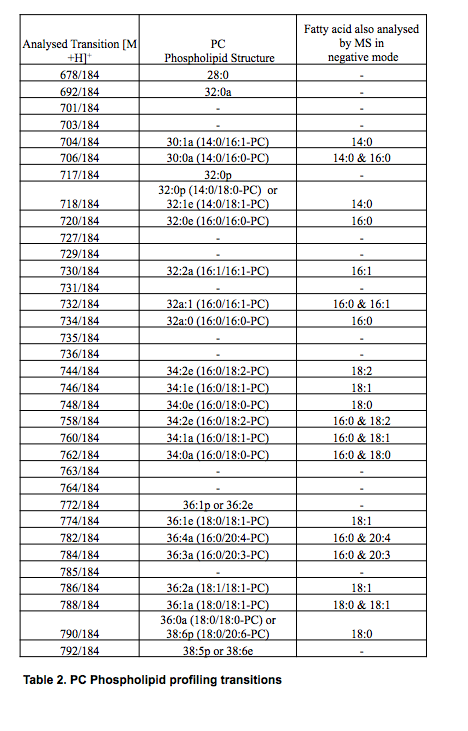


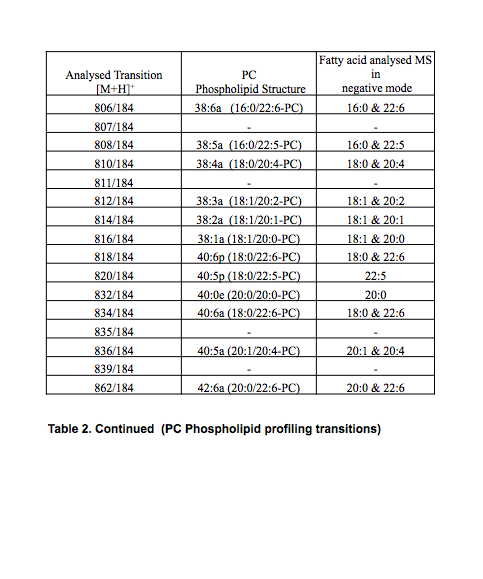


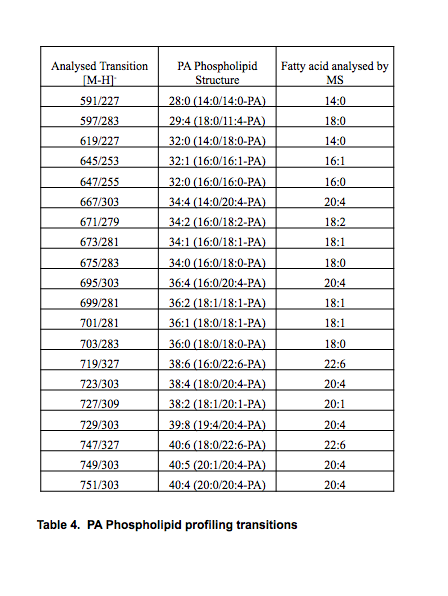


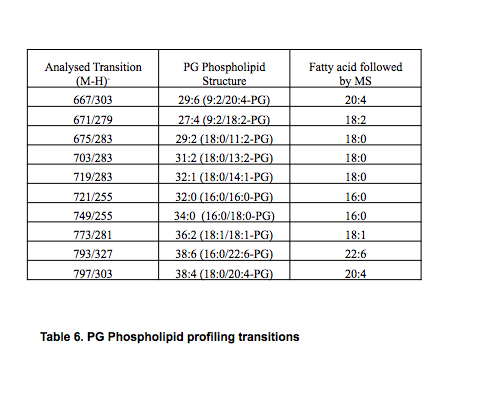

Supplement: Supplementary file 1 — Supplementary Data [file mmc1.docx]

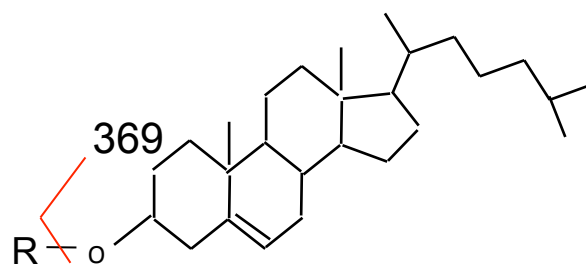

R=

|         |                      |
|---------|----------------------|
| 16:0-   | $[M+NH_4]^+ = 642.8$ |
| 16:1-   | $[M+NH_4]^+ = 640.8$ |
| 18:0-   | $[M+NH_4]^+ = 670.8$ |
| 18:1-   | $[M+NH_4]^+ = 668.8$ |
| 18:2-   | $[M+NH_4]^+ = 666.8$ |
| 18:2OH- | $[M+NH_4]^+ = 682.8$ |
| 20:4-   | $[M+NH_4]^+ = 690.8$ |
| 20:4OH- | $[M+NH_4]^+ = 706.8$ |
| 22:6-   | $[M+NH_4]^+ = 714.8$ |
| 22:6OH- | $[M+NH_4]^+ = 730.8$ |

**Scheme 1. Structures and  $m/z$  of cholesterol esters**

Supplement: Supplementary file 2 — Supplementary Data [file mmc2.pdf]
